# The Protective Role of Dormant Origins in Response to Replicative Stress

**DOI:** 10.3390/ijms19113569

**Published:** 2018-11-12

**Authors:** Lilas Courtot, Jean-Sébastien Hoffmann, Valérie Bergoglio

**Affiliations:** CRCT, Université de Toulouse, Inserm, CNRS, UPS; Equipe Labellisée Ligue Contre le Cancer, Laboratoire d’excellence Toulouse Cancer, 2 Avenue Hubert Curien, 31037 Toulouse, France; lilas.courtot@inserm.fr (L.C.); jean-sebastien.hoffmann@inserm.fr (J.-S.H.)

**Keywords:** dormant origins, replicative stress, replication timing, DNA damage, genome instability, cancer

## Abstract

Genome stability requires tight regulation of DNA replication to ensure that the entire genome of the cell is duplicated once and only once per cell cycle. In mammalian cells, origin activation is controlled in space and time by a cell-specific and robust program called replication timing. About 100,000 potential replication origins form on the chromatin in the gap 1 (G1) phase but only 20–30% of them are active during the DNA replication of a given cell in the synthesis (S) phase. When the progress of replication forks is slowed by exogenous or endogenous impediments, the cell must activate some of the inactive or “dormant” origins to complete replication on time. Thus, the many origins that may be activated are probably key to protect the genome against replication stress. This review aims to discuss the role of these dormant origins as safeguards of the human genome during replicative stress.

## 1. Introduction: Eukaryotic Origins and the Replication Program

Because of their large genomes, mammalian cells need thousands of replication forks, which initiate from replication origins, to ensure the complete duplication of their DNA within a specific time frame before they can divide. In human cells, the replication process takes about 10 h and involves the activation of roughly 30,000 replication origins. In normal replication conditions, replication origins are spread over about 100 kb of DNA, and only a single origin will be active within an individual DNA unit that we call a replicon. A coordinated group of adjacent replicons, “replicon cluster”, can be visualized as DNA replication foci [1]. Several studies, which compared replication timing (RT) and genome topology, suggested the term “replication domains” for replicons clustered inside large chromatin regions (~1 Mb), close to the size of one replication foci. They are located at discrete territories of the nucleus in the gap 1 (G1) phase and replicate at the same moment during the synthesis (S) phase [2,3,4]. At any given time of the S phase, about 10% of replicons are activated and replicate simultaneously [5]. In addition, the temporal activation of origins in a specific region of the genome correlates with a distinct pattern of replication foci as cells progress from early to late S phase. The sequential activation of potential origins within replication domains is thought to play a direct role in defining the S phase program or replication program. Temporal and spatial organization of DNA replication was adopted by metazoans cells to finely control the challenging goal of replicating the entire genome in a limited time and to overcome any obstacles that replication forks may encounter.

### 1.1. Origin Licensing and Firing

**Figure 1:**
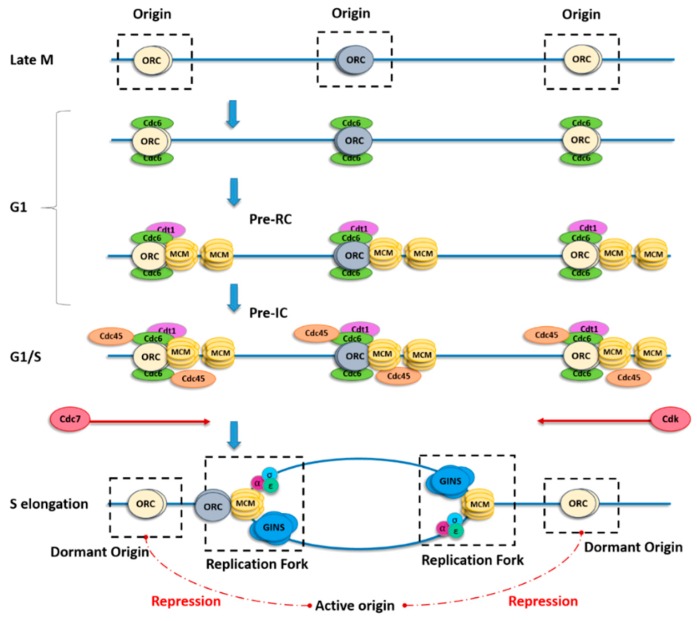
Scheme describing origin licensing and firing. In late mitosis (M), the origin recognition complex (ORC) binds to origins, thus determining where replication forks might initiate, and for the subsequent recruitment of cell division cycle 6 (Cdc6) and chromatin licensing and DNA replication factor 1 (Cdt1) in the gap 1 (G1) phase. Binding of both Cdc6 and Cdt1 is necessary, in turn, for recruitment of the minichromosome maintenance DNA helicase complex (MCM) to form the pre-recognition complex (Pre-RC). Each ORC has two Cdt1-binding sites, which may explain the cooperative loading of two MCM complexes per origin. The MCM pair remains catalytically inactive until the G1–synthesis (S) phase transition, when it is phosphorylated by both cyclin-dependent kinase (CDK) and Cdc7. Once the principal origin is fired, adjacent origins from the same replicon (flexible or dormant) are repressed (red dotted lines) by a yet unclear mechanism.

Complete and robust DNA duplication requires loading of minichromosome maintenance DNA helicase complex (MCM2–7) onto the replication origins. This step, called origin licensing, is restricted to the G1 phase of the cell cycle. A key initial step in origin licensing is the building of pre-recognition complex (Pre-RC) which starts with loading of the origin recognition complex (ORC) onto the chromatin. This ORC complex marks all potential origins providing spatial control of origin position. In higher eukaryotes, ORC binding sites were proven to be unrelated to DNA sequence, in contrast to other organisms such as yeast and bacteria [6,7]. It is currently assumed that multiple factors can characterize an origin, such as cytosine–phosphate–guanine (CpG) islands, G-quadruplexes, epigenetic marks, chromatin accessibility, sites of active transcription, or secondary DNA structures [8,9,10,11,12,13]. This is the reason why it is so difficult to identify metazoan replication origins. In the budding yeast *Saccharomyces cerevisiae*, a recent structural study [14] showed that two ORC molecules are required to ensure MCM2–7 complex loading onto the chromatin. During late mitosis and the G1 phase, ORCs bind cell division cycle 6 (Cdc6), which then interacts with chromatin licensing and DNA replication factor 1 (Cdt1) to allow loading of the six MCM subunits (MCM2–MCM7) and formation of the Pre-RC. The total amount of MCM complex does not change throughout the cell cycle, but the number of MCM complexes loaded onto DNA increases from telophase to the end of the G1–S phase transition. The final step of licensing requires the loading of Cdc45 and go-ichi-ni-san (GINS) onto the MCM complex to form the pre-initiation complex (Pre-IC). This complex requires the activities of the Dbf4-dependent kinase (DDK) and cyclin-dependent kinase (CDK) for its activation at the G1–S phase transition; then, the polymerases and other replication factors are recruited to allow origin firing (Figure 1).

During the first step of origin firing, the MCM pair slides along DNA by encircling the double helix. Recent papers proposed a switch of the MCM double hexamer from double-stranded DNA (dsDNA) to single-stranded DNA (ssDNA) mediated by N-tier ring movement, allowing the two helicases complexes to pass each other within the origin and permitting lagging-strand extrusion [15,16] (Figure 1). During the elongation step, excess MCMs that are not initiated are removed by the passage of the replication fork [17].

The cell must balance its need for sufficient origins to replicate the entire genome against the risk of re-replication of DNA in the S phase due to an excess of origins. Thus, the control of origin licensing is crucial. Repression of new origin licensing during the S phase is important to avoid re-replication, which can lead to aneuploidy, DNA double-strand breaks, gene amplification, and general genome instability [18,19,20]. DNA that is not replicated due to an insufficient number of origins or to replication fork stalling, by contrast, can also lead to genome instability and rearrangements if the DNA replication checkpoint is inactive or deficient [21,22,23].

### 1.2. Spatial and Temporal Organization of Replication Origins

Origin usage in eukaryotes is mainly dependent on two important factors: space and time. Replication origins fire at a defined time that remains the same among cell generations and is closely related to their spatial organization. Early replicating origins are mainly found in replication domains that are enriched in active epigenetic modifications and highly transcribed genes [24,25,26,27,28,29]. These chromosomal regions have a consequent amount of MCMs, providing potential origins that replicate early in the S phase [7,30]. Conversely, late replication occurs in origin-poor domains with low gene density, and enriched in heterochromatin hallmarks [29,31,32,33].

**Figure 2:**
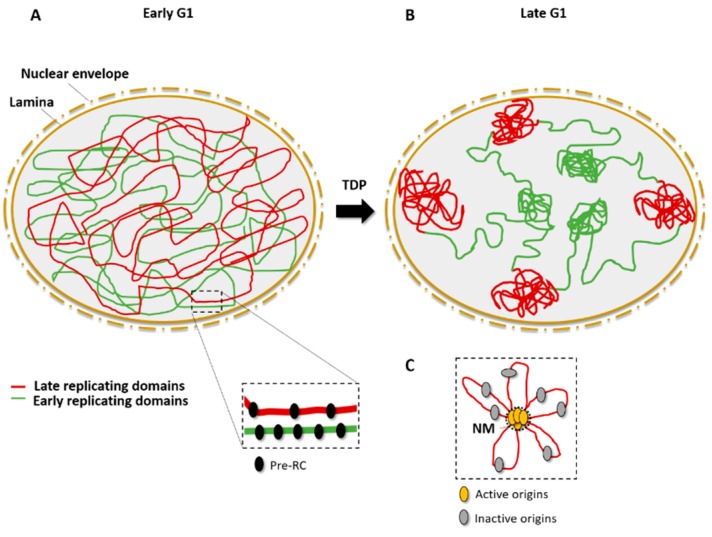
Spatial organization of origins and replication timing. (**A**) In the early G1 phase, Pre-RCs (black) are assembled on the chromatin and mark potential origins; early-replicating domains (green) and late-replicating domains (red) are disordered in the nuclear space. (**B**) After the timing decision point (TDP), in the late G1 phase, early-replicating domains are close to center of the nucleus whereas late-replication domains are associated with the lamina, close to the nuclear periphery. (**C**) Active origins (yellow) cluster in replication domains that are associated to the nuclear matrix (NM), leaving inactive (dormant or flexible) origins in DNA loops (gray).

Replication clusters are organized in the three-dimensional (3D) nuclear space, where early-replicating domains locate mainly at the center of the nucleus while late-replicating domains are found predominantly at the nuclear periphery (Figure 2B). Chromatin conformation mapping methods such as Hi-C are very powerful for visualizing the spatial organization of early- and late-replicating domains [34,35]. Replication domains are created by topological reorganization of the chromatin in nuclear space. In metazoans, the association of particular replication domains with sub-nuclear compartments determines their replication timing. The set-up of this compartmentalization occurs at a specific time of the G1 phase and is called the timing decision point (TDP) [36,37] (Figure 2).

Accumulating evidence indicates that DNA attachment to the nuclear matrix is important for the initiation of DNA replication [38,39,40,41,42]. The nuclear matrix permits the separation of chromosome territories and allows the formation of replication clusters [39]. The organization of replicon clusters might, thus, reflect chromatin looping to bring the origins from different replicons into a single domain and to exclude the flexible and/or dormant origins from this replication factory (Figure 2C). The cohesin complex may be a key player in chromatin looping because it was found to interact physically with the MCM complex and to be enriched at origin sites [43].

### 1.3. Techniques to Detect and Identify Origins

The first quantitative method for determining origin density in the genomes of bacteria and mammalians was DNA fiber autoradiography [44,45]. This time-consuming technique is now replaced by other assays, such as DNA combing or spreading, which label newly replicated DNA with nucleosides analogs, including bromo-, chloro-, and iododeoxyuridine, and visualize the newly replicated DNA by immunofluorescence microscopy using antibodies specific for the analog [46].

The use of next-generation DNA sequencing led to the discovery of tens of thousands of potential replication origins in the human genome. Several independent approaches were used that exploit the direct identification of DNA replication initiation intermediates. The first approach is based on the purification and quantification of short nascent strands (SNS) of DNA [26]. In this method, 1.5–2.5-kb nascent strands specific to replication origins are purified thanks to their resistance to λ-exonuclease digestion due to the incorporation, by the primase, of small RNA primers at their 5′ ends [47]. The exonuclease digests the large excess of broken genomic DNA that would generate a background signal if not correctly removed. These genome-wide SNS analyses showed that active origins often co-localize with transcription start sites (TSS) and are located in GC-rich regions, close to CpG islands or G-quadruplexes, confirming previous microarray hybridization results [24,25,48]. A second approach [29] is based on the sequencing of an early intermediate called the DNA replication bubble, which forms when two replication forks diverge from a single origin. The technique consists of fragmenting the replicating DNA via a restriction endonuclease, and then trapping the circular replication bubbles in agarose gel [29]. This so-called “bubble-seq” method led to the mapping of more than 100,000 origins in the human genome. A third genome-wide approach relies on sequencing purified Okazaki fragments (“OK-seq”) to determine replication fork polarity, which allows the identification of initiation and termination sites [49]. With this approach, between 5000 and 10,000 broad initiation zones of up to 150 kb were detected. These sites are mainly non-transcribed but often surrounded by active genes, and they contain a single randomly located initiation event. Finally, a fourth method for identifying metazoan replication origins is called initiation-site sequencing (“ini-seq”) [50]. In this method, initiation events are synchronized biochemically in a cell-free system in which newly replicated DNA, synthesized a few minutes after initiation, is directly labeled and subsequently immuno-precipitated. This original approach has the important advantage of allowing functional genome-wide studies of origin activation. As these approaches become more and more accurate and complementary to each other, they provide an increasingly large, novel dataset on the characteristics of replication origins.

### 1.4. Origin Flexibility, Dormancy, and Efficiency

The replication initiation program of metazoan cells is remarkably flexible, with many origins firing at disparate frequencies depending on the cell lineage. MCM complexes and all the components of the Pre-RC are loaded in excess onto the chromatin in the G1 phase to provide this flexibility. In addition to differences between cell lineages, origin flexibility is also observed within a cell population [42,51].

Very few origins are activated almost all the time; they are called “constitutive” origins [52]. The majority of origins do not initiate replication in all cell cycles; these are called “flexible” origins. Origins that are activated only when replication from adjacent origins is compromised are called “dormant” origins. Unlike constitutive and flexible origins, dormant origins are not detectable in whole-genome analyses. Inter-origin distances measured by whole-genome sequencing are shorter than those measured by single-fiber analyses. This discrepancy may be explained by the flexibility of origin choice within replicons [53], which might also help coordinate DNA replication with transcription [54,55] and other nuclear processes, such as DNA repair, in order to facilitate recovery when replication is compromised. Given that there is no DNA consensus sequence for metazoan origins and that there exists such a flexibility in establishing which potential origins are activated, one might wonder how initiation ever occurs accurately and at consistent origins [56].

There are currently two theories to explain how origins are selected. One relies on the idea of an origin decision point (ODP)—which occurs in the G1 phase, after the timing decision point—that determines which origins are activated during replication [57]. The second theory postulates increasing origin efficiency based on the random use of replication origins [58], with the idea that the efficiency of origin firing increases throughout the S phase as the replicative DNA polymerases recycle to new origins. Moreover, replication origin efficiency also depends on their location in the nucleus, epigenetic marks, and mainly on the amount of loaded MCM complexes [7,59,60] or nucleosome occupancy [61]. Chromosome architecture also plays an important role in the regulation of DNA replication origin localization and activation [62], although chromosomal loops and loop anchors are still poorly defined biochemically. Further studies using single-cell technologies will be required in the future to better understand the mechanism of origin choice.

## 2. Dormant Origin Activation in Response to Replicative Stress

### 2.1. The Notion of DNA Replication Stress

During DNA replication, the appearance of endogenous or exogenous sources of stress leads to replication forks slowing or stalling. Exogenous sources of stress comprise mainly genotoxic chemicals, and ultraviolet and ionizing radiation. Endogenous sources of stress that are considered to be barriers to replication include repetitive sequences, G-quadruplexes, telomeres, DNA–RNA hybrids, errors in the incorporation of ribonucleotides, collisions between replication and transcription machineries, compaction of chromatin, deregulation of origin activity, and reduction of the deoxyribonucleotide triphosphate (dNTP) pool. Some regions of the genome, such as early-replicating fragile sites (ERFSs) and common fragile sites (CFSs), are more prone than others to replicative stress. Moreover, evidence is emerging that constitutive activation or overexpression of oncogenes, such as Harvey rat sarcoma (HRas) and myelocytomatosis (c-Myc), are a potential source of replication stress [63]. These oncogenes promote replication initiation or origin firing, leading to an elevated risk of nucleotide pool depletion and/or increased collisions with transcription complexes [64,65]. This may explain why supplementing cancer cells with exogenous nucleosides helps decrease chromosomal instability [66].

The first consequence of replication stress is fork collapse, creating DNA single-strand breaks and/or double-strand breaks. These lesions must be resolved before cell division by repair mechanisms such as homologous recombination (HR), non-homologous end-joining (NHEJ). or micro-homology mediated end-joining (MMEJ). In normal cells, the ataxia telangiectasia mutated (ATM) and ataxia telangiectasia Rad3-related (ATR) checkpoint signaling pathways prevent cell division when the genome is damaged. When some proteins of the checkpoint pathway, for example p53, are mutated, the cell can divide despite the presence of DNA lesions (including breaks and unreplicated DNA), which may lead to chromosome fragmentation, rearrangements, and genomic instability [67,68,69,70].

### 2.2. The Discovery of Dormant Origins and Their Link to Replicative Stress

In 1977, J. Herbert Taylor [71] first described the firing of new origins in response to replication fork stalling during DNA replication in Chinese hamster ovary (CHO) cells, a finding that later suggested the existence of dormant origins. Moreover, several studies in a range of eukaryotes, including *S. cerevisiae*, *Xenopus laevis*, and human cells, demonstrated that MCM complexes are loaded onto DNA in a large excess when compared to the number of DNA-bound ORCs and the number of active replication origins [72,73,74,75,76,77]. It was later shown in *X. laevis* [78] and in human cells that this excess of MCM provides a reservoir of dormant origins, which are activated when replication forks are arrested by agents such as aphidicolin (APH) or hydroxyurea (HU) [79,80]. These studies also showed that depletion of MCM by small interfering RNAs leads to hypersensitivity to replication inhibitors due to the lack of dormant origins [79,80]. Moreover, checkpoint kinase 1 (Chk1) activation is required for firing of dormant origins within active replication clusters, as well as for repression of other replicons that are not yet active [81], suggesting a link between the DNA damage response and dormant origin activation. Indeed, in vertebrates, inactivation or depletion of various proteins involved in genome maintenance, such as ATR [82,83], Chk1 [84,85,86,87], Wee1 [88,89], bloom syndrome protein (BLM) [90], Claspin [91,92], breast cancer type 2 susceptibility protein (BRCA2), and Rad51 [93], slows replication forks and also increases the number of initiation events, at least in studies where initiation events were examined. This finding indicates a link between fork speed and the number of active origins, as we examine further below.

### 2.3. The Density of Active Origins Depends on Replication Fork Speed

Under normal conditions, dormant origins do not fire and are passively replicated by the fork coming from adjacent activated origins. Thus, it makes sense to assume that replication fork speed can be a regulator of active origin density. In two complementary studies on CHO cells [62,94], it was demonstrated that replication fork speed has a direct impact on the number of active origins. When the fork is slowed down by HU treatment, the density of active origins increases. In contrast, in conditions that accelerate fork speed (addition of adenine and uridine to the culture medium), fewer origins are active. These studies further showed that the cell starts compensating for the decrease in fork speed within half an hour of treatment by activating dormant origins, which are then able to change their status within the S phase. Regulation of the number of initiation events occurs at the level of individual clusters, consistent with the functional organization of origins into replicon clusters [95]. Another study demonstrated that, in the absence of Cdc7 or ORC1, replication forks progress more rapidly than in control cells and fewer origins fire [96], again suggesting that the number of active origins and the fork rate are interdependent. Similarly, using chemical inhibitors of origin activity (a Cdc7 kinase inhibitor) and of DNA synthesis (APH), a more recent study found that the primary effects of replicative stress on fork rate can be distinguished from those on origin firing [97]. Collectively, these results support the conclusion that the density of origin firing depends on fork speed and, thus, is affected by endogenous or exogenous replicative stress.

### 2.4. CFS Fragility Due to the Lack of Dormant Origins

CFSs play a major role in cancer initiation because of their instability in conditions of replication stress. CFSs were first described as gaps and constrictions in the metaphase chromosomes of human lymphocytes grown under mild replication stress conditions (i.e., a low dose of APH) [98]. These observations were since seen in other organisms and are very likely to be the consequence of under-replication and/or DNA breaks caused by replication stress [99,100].

Although CFSs have been known for over two decades, the cause of their fragility is still controversial [55,101]. CFS fragility was first linked to non-B DNA sequences, such as AT-rich sequences, which are able to adopt secondary structures, constituting barriers to replication forks [102,103,104,105]. Deletion of these sequences from some cancer cell lines does not prevent breaks at these loci [106,107,108], suggesting that DNA sequence is not the sole reason for the instability of CFSs. Genome-wide analysis of replication and DNA combing experiments found a paucity of replication origins within the core of CFSs [109,110] and an incapacity to activate additional origins in response to replicative stress [111]. This suggests that, in order to replicate these regions, the fork must pass through long stretches of DNA containing multiple non-B DNA conformation sequences, and that their fragility correlates with the absence of additional replication origin firing when replication is slowed down. Most CFSs correspond to long genes (>300 kb), which might increase the risk of collision between the transcription and replication machineries [112]. Although one study showed that the transcription of large genes does not systematically dictate CFS fragility [113], other studies found that replication stress induces locus- and cell-type-specific genomic instability at active, large transcription units corresponding to CFSs [114,115]. Moreover, it is thought that fragility of these sites result from entry into mitosis before their complete replication [116,117]. Taken together, these observations suggest that replication defects at fragile sites may be due to a low density of licensed origins or may reflect inefficient or delayed activation of replication forks under replication stress.

## 3. Regulation of Dormant Origins: A Passive or Active Mechanism?

### 3.1. Activation of Dormant Origins by a “Passive” Mechanism

It is currently not clear what drives the firing of dormant origins when forks are slowed down or inhibited. One first hypothesis could be that it does not involve an active mechanism, but occurs as a consequence of the stochastic nature of origin firing [18,79]. Dormant origins have a precise lap of time to fire before being passively replicated then inactivated by forks from adjacent origins. When fork progression is impeded, the replication at dormant origins is delayed and, therefore, they have an increased probability to fire. By means of computational modeling, a study showed that the same levels of dormant origin activation seen in vivo can be reproduced by a passive mechanism [118]. In this model, the mechanism relies simply on the stochastic nature of origin firing, without any need for additional regulatory pathways.

This simple theory can be sufficient to explain the activation of dormant origins in response to replicative stress. Nonetheless, it cannot be ruled out that dormant origins may also be regulated by active mechanisms, involving DNA damage response and other replication-related pathways.

### 3.2. Regulation of Dormant Origins by “Active” Mechanisms

#### 3.2.1. ATR/Chk1 Kinases as Modulators of Origin Activation

The inhibition of replication forks activates the DNA damage checkpoint kinases ATR–Chk1 and ATM–Chk2, which have many different functions, including stabilizing replication forks, delaying or blocking the progress of the cell cycle, and promoting DNA lesion repair [119,120,121]. It may seem surprising that, in response to replication stress, the cell can both activate dormant origins and suppress overall origin initiation; however, when replication forks stall, it makes sense that dormant origins should be activated in their vicinity and not elsewhere in the genome.

**Figure 3:**
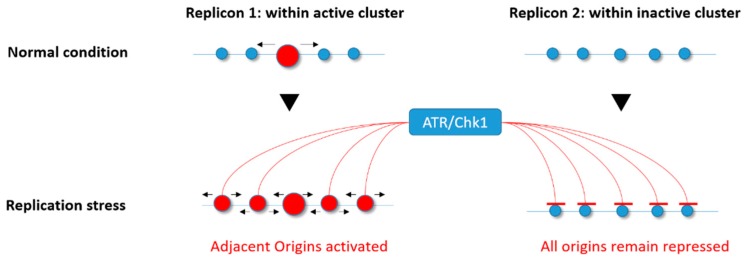
Ataxia telangiectasia Rad3-related (ATR)/checkpoint kinase 1 (Chk1) involvement in the differential regulation of origin firing under replicative stress. In response to replication stress, the ATR/Chk1 kinases allow the activation of dormant origins within active replicon clusters (active origin(s) in red) while repressing any firing within those that are not yet activated (inactive origins in blue).

In the normal S phase, Chk1 affects replication fork speed by inhibiting excess origin firing [23,85,86]. In response to low levels of replication stress induced by APH or HU, ATR and Chk1 impede the activation of new replicon clusters while allowing dormant origins to fire within those already activated and affected by the drug [79,81], thereby avoiding the deleterious impact of replication fork stalling (Figure 3). The mechanism responsible for this phenomenon is not yet elucidated, but one possibility is that ATR and Chk1 mildly reduce CDK levels, resulting in activation of fewer replication clusters [122]. Alternatively, Chk1 might directly inhibit the initiation process through an interaction with Treslin, which is required to stabilize Cdc45, GINS, and the MCM complex together with topoisomerase 2-binding protein 1 (TOPBP1) [123,124,125,126,127]. Moreover, a recent study found that an ATR inhibitor not only induced unscheduled origin firing, but also revealed another mechanism of origin regulation through a Cdc7-dependent phosphorylation of GINS [128]. Finally, a very recent study found that the ATR-activation domain of TOPBP1 is required to suppress origin firing during the S phase [129], further supporting an important role for the ATR–Chk1 pathway in regulating the activation of origins.

#### 3.2.2. Mannose Receptor C-Type 1 (Mrc1)/Claspin Is a Central Regulator of Origin Firing under Normal and Stressed Replication

*S. cerevisiae* Mrc1 and its metazoan ortholog Claspin are not only involved in the S phase checkpoint signaling pathway, but are also important components of replication forks. They interact with many factors known to function in or to regulate DNA replication, including MCM4, MCM10, ATR, Chk1, Cdc7, Cdc45, DNA polymerases α, δ, and ε, and proliferating cell nuclear antigen (PCNA) [130,131,132,133]. The presence of Mrc1/Claspin is necessary for normal DNA replication [91,92,134,135], probably by making a connection between the helicase components and replicative polymerases at the replication fork. Also, Claspin plays another role in the initiation of DNA replication in human cells during the normal S phase by recruiting Cdc7 to facilitate phosphorylation of MCM proteins [136]. It was recently discovered in yeast that Mrc1 has two crucial functions in regulating the firing of origins: a checkpoint independent-role to activate early-firing origins during normal replication, and a checkpoint dependent-function to inhibit late/dormant origins in the presence of HU [137].

#### 3.2.3. Fanconi Anemia Proteins in the Regulation of Dormant Origins

The role of the Fanconi anemia (FA) pathway in the DNA repair of interstrand cross-links (ICLs) was studied for many years. A clear model emerged describing that FA proteins orchestrate the interplay between multiple DNA repair pathways, including homologous recombination (HR) and translesion synthesis (TLS) [138,139,140]. However, treatment of cells with a low dose APH robustly activates the FA pathway, indicating a role of the FA proteins during DNA replication [141].

FA complementation group 1 (FANCI) was shown to be involved in dormant origin firing upon low replication stress through a FA pathway-independent mechanism [142]. FANCI associates with MCM3 and MCM5, localizes with replication origins, and acts as a regulator of DDK activity to allow the activation of the MCM2–7 helicase complex in response to mild replicative stress. In contrast, under high replicative stress, FANCI is phosphorylated by ATR. This phosphorylated form of FANCI negatively regulates dormant origin firing and activates replication fork restart/DNA repair that is FA-dependent. In this context, FA complementation group D2 (FANCD2), which is known as a close partner of FANCI, acts as a negative regulator of dormant origin firing [142].

Finally, FANCD2 was shown to facilitate replication of repeat-rich genomic regions such as CFSs by decreasing DNA–RNA hybrid accumulation, thus reducing the need for dormant origin firing [143].

#### 3.2.4. Rap1-Interacting Factor 1 (RIF1) Orchestrates Origins and Replication Timing 

RIF1 (Rap1-interacting factor 1) was first discovered in budding yeast as a telomeric chromatin-interacting protein required for the regulation of telomere length via its interaction with Rap1 [144,145]. It was then demonstrated in *S. cerevisiae* that RIF1 inhibits activation of the DNA damage checkpoint close to telomeres [146,147] and affects telomere replication timing [148]. Although the RIF1 protein is evolutionarily conserved, in metazoans, it was described not to play a specific role at telomeres, but rather to orchestrate the DNA double-strand break repair pathway and DNA recombination [149,150,151,152,153].

Further studies implicated RIF1 from the fission yeast *Schizosaccharomyces pombe* and mammalian RIF1 in regulating genome-wide DNA replication. *S. pombe* RIF1 binds selectively not only to telomeres, but also to specific regions of the genome where it may regulate the choice and timing of origin firing in late-replicating regions of chromosomes [154]. In RIF1-deficient cells, activation of dormant or late origins is concomitant with suppression of some active early-firing origins, indicating that RIF1 is a crucial player in the genome-wide origin activation program in *S. pombe*. In human cells, depletion of RIF1 results in increased early-S phase initiation events, loss of mid-S phase replication foci, and global changes in replication timing domain structures. Domains that normally replicate in the early S phase are delayed, whereas those that normally replicate in the late S phase are advanced [155]. Thus, replication timing is completely disturbed in the absence of RIF1. Another study observed that, in the absence of RIF1, the distance between origins is greater than in control cells during the normal S phase, and there are fewer dormant origins upon replication stress [156].

Also, RIF1 binds tightly to insoluble nuclear structures in late mitosis and the early G1 phase, and regulates chromatin-loop size [155]. Interestingly, RIF1 binding to consensus G-quadruplex-like sequences in fission yeast was identified [157]. These sequences tend to be near dormant origins, and the binding of RIF1 on these sites would allow their repression over a great distance. Overall, these findings indicate that RIF1, through its role in organizing higher-order chromatin architecture, is an essential regulator of replication timing.

Thus, the accumulating data suggest that, through its interaction with chromatin and nuclear structures, RIF1 plays an important role in the regulation of dormant origin availability not only in response to replicative stress, but also in normal conditions.

#### 3.2.5. Chromatin Loop Size Correlates with Dormant Origin Activation

The fluorescent DNA halo technique was essential for establishing the link between chromatin loops and replicon size [158], and for describing the importance of replicon remodeling events in *Xenopus* embryonic development [159]. Basically, the technique relies on cell permeabilization and soluble protein extraction, allowing supercoiled DNA loops to unroll around an insoluble scaffold, the nuclear matrix. Those structures called DNA “halos” can be visualized by 4′,6-diamidino-2-phenylindole (DAPI) fluorescent staining. Active origins are in or near the nuclear matrix, whereas dormant/inactive origins are in the DNA loops [160] (Figure 2C).

Using the fluorescent DNA halo technique, one study [62] observed a strict correlation between dormant origin activation at a given S phase and reduced chromatin loop size in the next G1/S phase. Combining the DNA halo experiment with fluorescent in situ hybridization (FISH) using a probe targeting the highly amplified adenosine monophosphate deaminase 2 (AMPD2)-specific locus in CHO cells, they demonstrated that, in response to replication stress, activation of dormant origins relocates this locus toward the nuclear matrix.

Cohesin also influences the size of interphase chromatin loops since its absence results in longer chromatin loops due to a limited origin usage [43], showing that, independently of the effect of cohesin acetylation on replication fork progression [161], this structural protein is present at origins and impacts their activity. Finally, chromatin loop size increases in RIF1-depleted cells [155], suggesting that the RIF1 protein is required for proper chromatin loop formation, as already mentioned above.

## 4. Dormant Origin Deficiency, Genome Stability, and Pathologies

### 4.1. MCM Mutants and Dormant Origins in Mice

Homozygosity for a null allele of any of the six *Mcm* genes in mice (*Mcm2*–*7*) causes embryonic lethality [162,163,164], consistent with the evidence that these *Mcm* genes are essential for DNA replication. Only hypomorphic alleles such as *Mcm4^Chaos3^* and *Mcm2^IRES-CreERT2^* (IRES, internal ribosome entry site; ERT2, estrogen receptor 2) result in mice that are viable into adulthood. The *Mcm2^IRES-CreERT2^* allele expresses a tamoxifen-inducible Cre recombinase (CreERT2) inserted into the 3′ untranslated region (UTR) of the endogenous *Mcm2* locus, which reduces the expression of *MCM2* by 65% when compared to wild-type cells [165]. The *Mcm4^Chaos3^* allele produces an MCM4 protein with a Phe345Ile mutation, which does not affect the helicase activity of the MCM complex in vitro, but does reduce the efficiency of its assembly [164].

Surprisingly, mouse embryonic fibroblasts (MEFs) from *Mcm4^Chaos3^* mice also have a reduced MCM7 protein level in addition to MCM4 [164]. Moreover, immortalized homozygous *Mcm4^Chaos3^* cells display less stable association of MCM2–7 at replication forks compared to wild-type cells [166]. Finally, *Mcm4^Chaos3/Chaos3^* MEFs exhibit about a half reduction in chromatin bound MCM2–7 that causes a lower ability to activate dormant origins in response to treatment with low doses of APH [162,167].

Mice with only one-third of the normal MCM2 level were shown to develop lymphomas at a very young age, and have diverse stem cell proliferation defects. Similarly to *Mcm4^Chaos3^*, these mice also have 27% less MCM7 protein than wild-type mice. Moreover, *Mcm2^IRES-CreERT2^* cells exhibit decreased replication origin usage due to lower dormant origin availability even in the presence of HU, as demonstrated by DNA combing experiments [165,168].

Hence, these two mouse models are close phenotypically, showing dormant origin deficiency due to reduced levels of loaded MCM onto the chromatin. Even in an unchallenged S phase, the inability to activate dormant origins leads to accumulation of stalled replication forks that reach mitosis and interfere with chromosome segregation. Both phenotypes lead to improper chromosome stability and premature tumorigenesis, with several differences in the latency of disease development [165,166,168,169].

### 4.2. MCM Mutants and Dormant Origins in Stem/Progenitor Cells

The fact that *Mcm2* expression has a global effect on cell proliferation within many tissues might explain why the majority of *Mcm2^IRES-CreERT2^* mice develop tumors and display a range of additional hallmarks of age-related disorders. A study that set out to determine the effect of *Mcm2* deficiency observed an approximately threefold reduction in the level of neurogenesis within the sub-ventricular zone in *Mcm2^IRES-CreERT2^* mouse brains [165], fewer stem cells in intestinal crypts and in skeletal muscle, and a modest increase in DNA damage.

Consistent with the conclusion that *Mcm* mutants affect stem cells, neural stem-cell progenitors in *Mcm4^Chaos3/Chaos3^* mouse embryos display a high level of Chk1 activation, increased phosphorylated H2A histone family X (γH2AX) and p53-binding protein 1 (53BP1) foci, an accumulation in the G2–M phase, and more apoptosis, resulting in a reduced ability to form neurospheres in vitro [170]. The renewal of stem cells in the brain appears to be normal, but their ability to differentiate into intermediate progenitors is highly reduced due to an increase of apoptotic cells in the sub-ventricular and intermediate zones [170].

These observations suggest that normal expression of MCM complex proteins is essential for stem/progenitor cell function by reducing the risk of replication-associated genome instability, an idea that was supported by two other studies. One demonstrated that human embryonic stem cells, which have a remarkably short G1 phase, load MCM onto chromatin very rapidly when compared to differentiated cells, in order to have a similar total amount of loaded MCM at the G1–S phase transition [171]. In the second study, hypomorphic expression of the origin licensing factor MCM3 in mouse reduced the number of licensed origins and affected the function of hematopoietic stem cells, as well as the differentiation of highly proliferative erythrocyte precursors, thus demonstrating that the rate of MCM loading is crucial for correct organism development [163]. These observations suggest that hematopoietic progenitors are exceptionally sensitive to replication stress, and that they must license an excess of origins to ensure their correct differentiation and function.

Intriguingly, aging hematopoietic stem cells suffer from replication stress even in wild-type mice. This might be due to the fact that old stem cells have reduced expression of MCM complex proteins, resulting in reduced numbers of dormant origins and, as a consequence, more chromosome instability and cell-cycle defects [172].

### 4.3. Consequences of Limited Licensing and Firing in Humans

A mutation in the *Mcm4* gene, which results in a truncated form of this protein lacking the N-terminal serine/threonine-rich domain, was identified in a group of patients with a syndrome including growth delay, natural killer cell deficiency, adrenal insufficiency, and genome instability [173,174,175]. This truncated form of MCM4 does not affect MCM complex loading [174]. Nevertheless, immortalized fibroblasts from these patients have a high level of chromosome breakage and defects in cell-cycle progression, and they are sensitive to low doses of APH [174], suggesting that the N-terminal amino acids of MCM4 protein are involved in the maintenance of genome integrity during replication. Further studies will be necessary to elucidate the mechanism via which normal MCM4 ensures genome maintenance. One possibility is the role of MCM4 phosphorylation in the checkpoint response, where it was shown that the N-terminal domain of MCM4 has a crucial role. In unperturbed replication, this domain exerts an inhibitory effect on replication initiation, and this inhibitory effect is relieved upon its phosphorylation by DDK. However, in the context of replication stress, this N-terminal phosphorylation by DDK becomes a prerequisite for proper checkpoint activation [176].

Another disease that appears to involve defective replication origin licensing is Meier–Gorlin syndrome (MGS), an autosomal recessive primordial dwarfism syndrome characterized by pre- and post-natal impaired growth. Several studies identified marked locus heterogeneity in this syndrome including mutations in five genes encoding components of the Pre-RC: *Orc1*, *Orc4*, *Orc6*, *Cdt1*, and *Cdc6* [177,178]. The molecular and cellular phenotypes include impaired licensing, altered S phase progression, and proliferation defects, which partially overlap with the phenotypes due to MCM mutations, except for chromosomal instability, and an increased predisposition to cancer. Nonetheless, MGS mutations in *Orc1* and *Orc6* can cause quite a significant reduction in MCM loading and replication origin licensing [177,179,180].

Mice and human phenotypes caused by mutations in the licensing system illustrate our limited understanding of what happens to cells when the DNA replication program is compromised. For example, the threshold value for the number of licensed origins needed to activate the licensing checkpoint is still not known, nor whether this value varies between cell types.

## 5. Conclusion and Prospects

Dormant origins are now recognized as an important safeguard against under-replication of the genome, thus ensuring genome maintenance. Activation of dormant origins plays a central role in the rescue of stalled forks in the context of replicative stress, contributing to the complete replication of the DNA. The interactions between dormant origins and other fork restart mechanisms (such as TLS) are mostly unknown, even though some links with DNA damage checkpoint or FA pathways are becoming evident. What determines whether the cell activates dormant origins or induces these other mechanisms in response to fork stalling still remains to be investigated.

How replicon clusters are activated at the molecular level remains unclear, although we know that origin activation is regulated by both Chk1 and CDKs. The RIF1 protein might be the most interesting factor in this process since it is present both at the replication fork and at replication origins, where it plays a role in the DNA damage response, as well as in replication timing.

**Figure 4:**
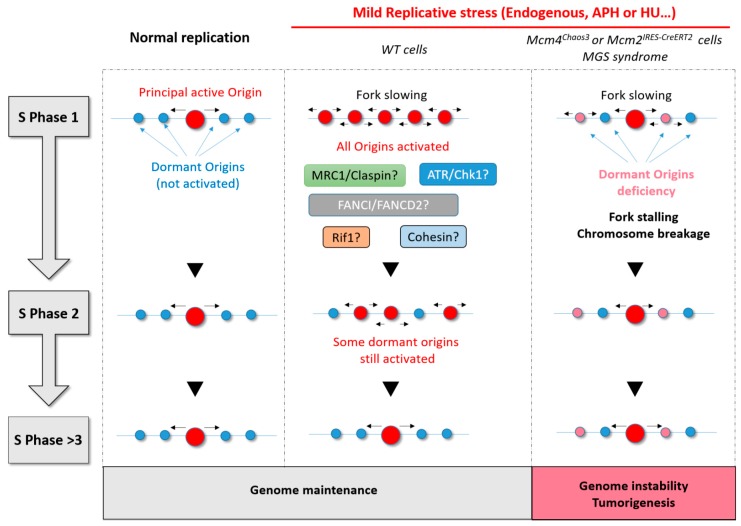
Summary diagram showing the importance of dormant origin activation in response to replicative stress. During normal replication, only the principal origin is activated. If there is no replicative stress, this same principal origin is also activated in the next S phase. Under conditions of mild replicative stress, adjacent or dormant origins fire to compensate for fork slowing and to allow complete replication on time. Many proteins (ATR/Chk1, mannose receptor C-type 1 (Mrc1)/Claspin, Fanconi anemia complementation group 1 (FANCI)/ Fanconi anemia complementation group D2 (FANCD2), and Rap1-interacting factor 1 (RIF1)) are thought to be involved in the regulation of dormant origins under mild replicative stress. RIF1 and Cohesin are two good candidates to explain the persistence of some origin activation in the next S phase. Finally, when cells have few origins or a deficiency in dormant origins, replicative stress leads inevitably to fork stalling, DNA breaks, and genomic instability with a consequent risk of tumorigenesis.

The study that found a direct correlation between origin activation and chromatin loop size [62] also reported that origins located near the anchorage sites of chromatin loops are preferentially activated in the S phase of the following cell generation (Figure 4). This suggests that cells respond to changes in fork dynamics by adapting origin usage in the next cell cycle, in addition to their rapid response of origin activation. It appears that cells can adapt to grow under conditions of fork slowing by increasing the efficiency of some origins that are usually dormant in normal growth conditions.

Perhaps most exciting is the prospect that the regulation of dormant origins might be different in cancer cells to that in normal cells. MCM complex proteins are often misregulated at the early stage of cancer [18,181,182], and tumor cells are more sensitive to replicative stress when they have a reduced origin licensing capacity [183]. Mice hypomorphic for *Mcm* gene expression demonstrate the real importance of dormant origins, but any link with spontaneous cancer development remains to be determined to see whether this information can be useful to deal with anti-cancer molecules more accurately.

## Abbreviations

53BP1	p53-Binding protein 1
APH	Aphidicolin
ATM	Ataxia telangiectasia mutated
ATR	Ataxia telangiectasia and Rad3-related protein
BLM	Bloom syndrome RecQ like helicase
BRCA2	Breast cancer 2
Cdc45	Cell division cycle protein 45
Cdc6/7	Cell division cycle 6/7
CDK	Cyclin-dependent kinase
Cdt1	Chromatin licensing and DNA replication factor 1
CFS	Common fragile site
Chk1	Checkpoint kinase 1
Chk2	Checkpoint kinase 2
CHO	Chinese hamster ovary
c-Myc	Myelocytomatosis
DDK	Dbf4-dependent kinase
DNA	Deoxyribonucleic acid
dNTP	Deoxyribonucleotides
ERFS	Early-replicating fragile site
FANCI/D2	Fanconi anemia complementation group I/D2
FISH	Fluorescence in situ hybridization
GINS	Go-ichi-ni-san
HR	Homologous recombination
HRAS	Harvey rat sarcoma
HU	Hydroxyurea
ICL	Interstrand cross-link
MCM	Minichromosome maintenance
MGS	Meier–Glorin syndrome
MMEJ	Micro-homology mediated end-joining
Mrc1	Mannose receptor C-type 1
NHEJ	Non-homologous end-joining
ODP	Origin decision point
ORC	Origin recognition complex
PCNA	Proliferating cell nuclear antigen
Pre-IC	Pre-initiation complex
Pre-RC	Pre-recognition complex
RIF1	Rap1-interacting factor 1
RNA	Ribonucleic acid
RT	Replication timing
SNS	Short nascent strand
SVZ	Sub-ventricular zone
TDP	Timing decision point
TOPBP1	Topoisomerase 2-binding protein 1
TLS	Translesion synthesis
TSS	Transcription start sites
